# A subset of low-grade B cell lymphomas with a follicular growth pattern but without a *BCL2* translocation shows features suggestive of nodal marginal zone lymphoma

**DOI:** 10.1007/s12308-015-0259-y

**Published:** 2015-09-02

**Authors:** Michiel van den Brand, Olga Balagué, Patricia H. J. van Cleef, Patricia J. T. A. Groenen, Konnie M. Hebeda, Daphne de Jong, J. Han J. M. van Krieken

**Affiliations:** Department of Pathology, Radboud University Medical Center, box 9101, 6500 HB Nijmegen, The Netherlands; Hematopathology Section, Laboratory of Pathology, Hospital Clínic, Institut d’Investigacions Biomèdiques August Pi i Sunyer, University of Barcelona, Barcelona, Spain; Department of Pathology, VU Medical Centre, Amsterdam, The Netherlands

**Keywords:** Follicular lymphoma, Nodal marginal zone lymphoma, Low-grade B cell lymphoma, BCL2

## Abstract

In our consultation practice, it was noted that many cases that were considered to represent follicular lymphoma (FL) without a *BCL2* translocation were ultimately classified as nodal marginal zone lymphoma (NMZL). This study set out to define recurrent morphological features of these cases. Thirty-three low-grade B cell lymphomas without a *BCL2* rearrangement were studied for recurrent morphological features. These features were then applied on 20 randomly selected cases to verify if these criteria are able to distinguish between lymphomas with and without a *BCL2* rearrangement, assigning them to one of five categories ranging from “certain FL” to “certain NMZL.” Highly recurrent morphological features were noted in the lymphomas without a *BCL2* rearrangement, which were strongly overlapping with the morphological features of NMZL. All six cases that were assigned to the category of certainly FL or most likely FL indeed harbored a *BCL2* rearrangement, whereas all 12 cases assigned to the category of most likely NMZL or certain NMZL had no *BCL2* break. Of the two cases in the ambiguous category, one had received a final diagnosis of FL and the other of NMZL. This study raises the hypothesis that a subset of low-grade B cell lymphomas with a follicular growth pattern but without a *BCL2* translocation actually represents NMZL. This is at present difficult to prove, because no gold standard is available to differentiate between NMZL and FL without a *BCL2* rearrangement, so further investigations are needed.

## Introduction

Follicular lymphoma (FL) is a low-grade B cell lymphoma that has a translocation involving *BCL2* in up to 90 % of cases, resulting in BCL2 upregulation. It usually has a follicular growth pattern with expression of germinal center markers (e.g., CD10 and BCL6). In most cases, a diagnosis of FL is rather straightforward and can be made by morphology and immunohistochemistry alone. However, in the absence of characteristic morphology, typical immunophenotype, or in the absence of a *BCL2* translocation, the diagnosis becomes more difficult. An important difficulty results from the morphological and immunohistochemical overlap between FL and nodal marginal zone lymphoma (NMZL) with a nodular/follicular growth pattern. NMZL is currently defined as “a primary nodal B cell neoplasm that morphologically resembles lymph nodes involved by MZL of extranodal or splenic types but without evidence of extranodal or splenic disease” [1]. The main problem is the lack of positive markers for NMZL, in fact making this a diagnosis of exclusion. Previous case reports and small studies indicate that NMZL may easily be misdiagnosed, especially when there is extensive follicular colonization [2–4]. The follicles that are colonized result in a nodular architecture with partial positivity for CD10 and BCL6 due to the pre-existent germinal center cells that are in the same compartment as the tumor cells and acquisition of BCL6 expression by the colonizing neoplastic cells. The colonizing NMZL cells are usually BCL2 positive which causes the suggestion of BCL2-positive follicles.

In routine diagnostic practice, it was noted that lymphomas that lack a *BCL2* translocation, but display BCL2-positive follicles at low power, were often considered to represent FL. However, careful study of BCL2 at high power in these cases often showed negative cells as a sign of follicular colonization, suggesting a diagnosis of NMZL rather than FL. In this study, we set out to substantiate the hypothesis that a subset of lymphomas that are initially classified as FL without *BCL2* translocation (translocation-negative FL), actually represent NMZL.

## Material and methods

### Case selection

Fifty-six primary nodal B cell lymphomas, both from in-house patients (*n* = 21) and consultation cases (*n* = 35), were retrieved from the archive of the Department of Pathology of the Radboud University Medical Center (Nijmegen, the Netherlands). These were selected based on the fact that they had been tested for a *BCL2* translocation by fluorescence in situ hybridization (FISH) between 2004 and 2010. During this period, FISH was used in diagnostic practice in all cases with a differential diagnostic consideration of FL and NMZL.

Three cases that had originally been classified as NMZL were excluded because they were considered by the study group to represent chronic lymphocytic leukemia, diffuse large B cell lymphoma, and follicular lymphoma grade 3B, respectively. The study group therefore consisted of 53 cases, 33 without a *BCL2* rearrangement and 20 with a *BCL2* rearrangement.

### Immunohistochemistry

Immunohistochemistry was performed in routine diagnostic practice according to standard laboratory procedures. Briefly, 4-μm sections were cut from formalin-fixed paraffin-embedded (FFPE) tissue, dried, deparaffinized, rehydrated, and subjected to heat-mediated antigen retrieval. Specific retrieval procedures for each antigen are indicated in Table 1. Subsequently, endogenous peroxidase was blocked after which the primary antibody (Table 1) was applied for 1 h at room temperature (RT). The secondary antibody (Powervision poly-HRP-anti MS/Rb/Rt, Immunologic, Klinipath, Duiven, the Netherlands) was then applied for 30 min at RT after which visualization was performed using bright 3,3′-diaminobenzidine. Hematoxylin was used as a nuclear counterstain.

**Table 1:** Overview of antibodies

Antigen	Manufacturer	Dilution	Retrieval
BCL2	Dako (clone 124)	1:160	Citrate pH 6.0, 10 min 96 °C
BCL6	Novocastra (Clone LN22)	1:40	Citrate pH 6.0, pressure cooker
CD5	Menarini (Clone 4C7)	1:80	Citrate pH 6.0, 10 min 96 °C
CD10	Monosan (Clone 56C6)	1:20	Citrate pH 6.7, 30 min 100 °C
CD20	Thermo Scientific (Clone L26)	1:300	Citrate pH 6.0, pressure cooker
CD23	Thermo Scientific (Clone 1B12)	1:10	Citrate pH 6.0, 10 min 96 °C
CD79a	Dako (Clone JCB117)	1:400	Citrate pH 6.7, 30 min 100 °C
Cyclin D1	Immunologic (Clone SP4)	1:80	Citrate pH 6.7, 30 min 100 °C
Ki-67	Dako (Clone MIB1)	1:200	Citrate pH 6.0, 10 min 96 °C

### Fluorescence in situ hybridization

FISH was performed on 4-μm sections from FFPE tissue that were dried, deparaffinized, rehydrated, and heated for 10 min in sodium citrate (pH 6.0). Slides were rinsed in 0.01 M hydrochloric acid before 15 min of pepsin digestion. After three rinses of 0.01 M hydrochloric acid and 5 min of fixation in 1 % formaldehyde in PBS, sections were dehydrated and dried after which the probe was added (*BCL2* DNA probe, Dako, Y5407, Glostrup, Denmark; *BCL6* DNA probe, Dako, Y5408, Glostrup Denmark). After 5 min of denaturation at 82 °C, hybridization was performed overnight at 45 °C in a Dako S2451 hybridizer (Dako). Sections were then dehydrated, dried, and mounted using medium containing DAPI as a nuclear counterstain (Vectashield, Vector Laboratories, Burlingame, CA). Stained sections were assessed with a Leica DM 4000B fluorescence microscope.

For *BCL2* FISH, all cases were evaluated and documented by the technician (PvC), with subsequent confirmation by one of the hematopathologists (KH, JvK). FISH for *BCL6* was scored by a single observer (MvdB). Split signals in 20 % of cells or more were regarded as positive, although in most positive cases, the amount of positive cells was far above 20 %.

### Morphological evaluation

Based on cases lacking a *BCL2* translocation, firstly (immuno)morphological criteria were set for the diagnosis of NMZL (JVK, OB).

Subsequently, these criteria were applied on 20 of the 53 cases (including 7 cases with and 13 without *BCL2* translocation) by four hematopathologists (JvK, OB, DdJ, KH) at a multi-headed microscope with all histochemical and immunohistochemical stainings but without clinical information or knowledge of *BCL2* translocation status. These cases were scored on a 5-point scale: certainly FL (1), most likely FL (2), ambiguous (3), most likely NMZL (4), or certainly NMZL (5).

## Results and discussion

This study followed on the recognition that in consultation practice, lymphomas that lack a *BCL2* translocation, but display BCL2-positive follicles at low power, may easily be misdiagnosed as FL. However, after careful evaluation of H&E morphology and BCL2 immunohistochemical staining at high power, NMZL could be recognized. From this observation, we hypothesized that a subset of lymphomas that are initially classified as translocation-negative FL are actually better classified as NMZL.

To investigate this, 53 low-grade B cell lymphomas in the spectrum of the differential diagnosis of FL and NMZL were selected. Of these, 20 did show a *BCL2* rearrangement with a FISH split probe and were classified as FL. Thirty-three cases did not show a *BCL2* rearrangement. Table 2 shows the diagnoses that were made at the time the biopsy was taken and the final diagnosis, which was established during this study, for all lymphomas lacking a *BCL2* translocation.

**Table 2:** Diagnosis of cases without a *BCL2* break

Referring center diagnosis	Referral center diagnosis	Final diagnosis within this study	Number
Primary cases (*n* = 13)			
	NMZL	NMZL	9
	FL	NMZL	4
Referral cases (*n* = 20)			
NMZL	NMZL	NMZL	1
FL	NMZL	NMZL	7
FL	FL	NMZL	2
FL/NMZL	NMZL	NMZL	2
Low-grade B	NMZL	NMZL	3
Low-grade B	FL	NMZL	2
Reactive	NMZL	NMZL	1
Reactive	FL 3B	NMZL	1
CLL/SLL	NMZL	NMZL	1

*NMZL* nodal marginal zone lymphoma, *CLL*/*SLL* chronic lymphocytic leukemia/small lymphocytic lymphoma, *FL* follicular lymphoma grade 1–2, *Low*-*grade B*, low-grade B cell lymphoma, *FL 3B* follicular lymphoma grade 3B

**Fig. 1:**
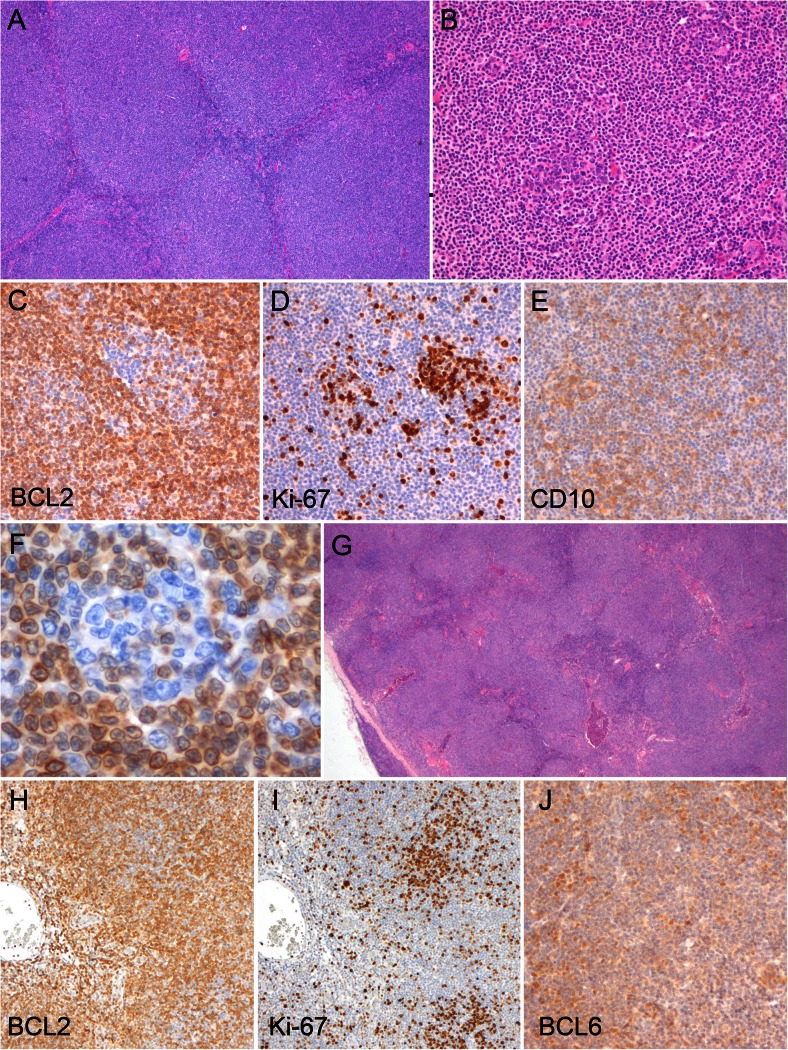
Morphological features of cases without *BCL2* translocation. **a**–**f** Lymph node showing completely effaced architecture with a nodular appearance on low power (**a**). On medium power (**b**), residual follicles are seen which are BCL2 negative (**c**) and show a high proliferative index (**d**). CD10 (**e**) is expressed not only in the residual follicles but also in the surrounding neoplastic cells. BCL2 staining shows blasts without BCL2 expression at high power (**f**). This case was classified as “certainly NMZL” based on morphological features. **g**–**j** Another lymphoma with nodular architecture on high power (**g**). BCL2 immunohistochemistry shows poorly defined areas with BCL2-negative cells (**h**). In these areas, a high proliferative index is present, indicated by frequent Ki-67-positive cells (**i**). BCL6 shows diffuse positivity with heterogeneous intensity (**j**). This case was classified as “most likely NMZL” based on morphological features

In a first effort, the morphological features in the translocation-negative group were evaluated. The following features were observed to be highly recurrent (Fig. 1): (1) effaced architecture of the lymph node by a small B cell proliferation with a follicular, marginal zone, or diffuse growth pattern; (2) centrocytic or “CLL-like” small cell morphology with intermingled centroblasts; (3) an immunophenotype of a mature B cell (CD20 and CD79a positive) with BCL2 positivity and lacking expression of cyclin D1 and CD5; (4) The cases with a follicular/ nodular growth pattern showed follicular colonization; the presence of both neoplastic and reactive cells in a follicle. For the assessment of follicular colonization, BCL2 and Ki67 immunohistochemistry were carefully evaluated. For BCL2, positive cells (the colonizing lymphoma cells) could be recognized among negative cells (the pre-existent cells) at high power. For Ki67, clusters of highly proliferative cells characterized follicles being colonized, whereas truly neoplastic follicles were characterized by a relatively low proliferative index. Expression of CD10 in the BCL2-positive cells was observed only rarely (in 5 out of 21 cases), with mostly weak staining. In 3 out of 16 cases, weak BCL6 expression was observed in BCL2-positive cells.

The strong overlap between the morphological features in lymphomas lacking *BCL2* rearrangements in this series and the morphological features of NMZL is in line with our hypothesis that a subset of lymphomas that are initially classified as translocation-negative FL actually have features of NMZL.

To verify if the criteria defined above were able to recognize lymphomas without a *BCL2* rearrangement, they were applied to 20 cases that were selected randomly from the total of 53 cases. These 20 cases were reviewed without knowledge of clinical features or the presence of a *BCL2* translocation and assigned to one of five categories ranging from “certain FL” to “certain NMZL,” as indicated in Table 3. All six cases that were assigned to the category of certainly FL or most likely FL indeed harbored a *BCL2* rearrangement, whereas all 12 cases assigned to the category of most likely NMZL or certain NMZL had no *BCL2* break. Of the two cases in the ambiguous category, one had received a final diagnosis of FL and the other of NMZL.

As indicated in Table 2, many cases (42 %) that received a final diagnosis of NMZL in this study were at some point considered to be FL. Only one *BCL2* translocation-negative case was ultimately classified as follicular lymphoma. Because the morphological criteria for the absence of a *BCL2* rearrangement showed strong overlap with the morphological criteria for NMZL, and because the large majority of lymphomas without a *BCL2* rearrangement were ultimately diagnosed as NMZL, these results suggest that a subset of lymphomas that are classified as translocation-negative FL are actually NMZLs. Although the results are suggestive, establishing a final proof for this hypothesis is currently very difficult if not impossible, because specific criteria for NMZL are lacking. The fact that some lymphomas without a *BCL2* rearrangement showed expression of the germinal center markers CD10 and/or BCL6 is an argument in favor of a diagnosis of FL. However, the morphological features outlined above that were present in these lymphomas argue against this. Also, expression of CD10 and BCL6 has been reported in NMZL [2, 5, 6]. In our series, CD10 was positive in the BCL2-positive cells in 5 out of 21 (24 %) cases, with mostly weak staining. BCL6 showed weak staining in the BCL2-positive cells in 3 out of 16 (19 %) cases. The most important feature to be recognized in this setting is follicular colonization. We determined the presence of follicular colonization by combining BCL2 and Ki67 staining. Although colonized follicles seemed to be BCL2-positive on low power examination, at high power, both BCL2-positive (the colonizing lymphoma cells) and BCL2-negative (the pre-existent cells) cells were recognized. A high proliferative index, as indicated by a high proportion of Ki67-positive cells also characterized follicles being colonized, whereas truly neoplastic follicles were characterized by a relatively low proliferative index. Finally, in many cases of FL, the BCL2 expression is stronger than that of remaining mantle zone B cells and T cells.

**Table 3:** Application of NMZL criteria

Morphological score	Number	*BCL2* break	Final diagnosis	
		*n*	*n* (%)	
1: certainly FL	1	1	FL	1
			NMZL	0
2: most likely FL	5	5	FL	5
			NMZL	0
3: ambiguous	2	1	FL	1
			NMZL	1
4: most likely NMZL	2	0	FL	0
			NMZL	2
5: certainly NMZL	10	0	FL	0
			NMZL	10

*FL* follicular lymphoma, *NMZL* nodal marginal zone lymphoma

FLs without a *BCL2* rearrangement might have a rearrangement involving *BCL6*. We studied 22 of the cases without a BCL2 rearrangement for the presence of a rearrangement involving *BCL6*, using a dual-color break-apart probe locating to *BCL6*. In 5 out of 22 cases (23 %), a rearrangement of *BCL6* was identified. Of these 5 cases with a *BCL6* rearrangement, one was weakly positive for CD10, one was positive for CD10 and weakly positive for BCL6, one was weakly positive for BCL6, and two were negative for both CD10 and BCL6. In summary, 3 out of 5 (60 %) cases with a *BCL6* rearrangement showed expression either CD10 or BCL6 versus 2 out of 12 (17 %) cases without a *BCL6* rearrangement. Although these results suggest some enrichment for germinal center marker expression in cases with a *BCL6* rearrangement, the difference between these groups was not statistically significant (*p* = 0.117 with Fisher’s exact test). This finding is of potential interest, but a definitive conclusion would require the study of additional cases.

Cases were included in this study when they had been tested for a *BCL2* translocation with FISH. In general, *BCL2* FISH is not performed on cases that are considered to be straightforward FL by morphology and immunohistochemistry, resulting in a bias toward atypical cases in our study. This also means that the group of “straightforward” FLs, that was not included in this study, might contain *BCL2* translocation-negative cases, and we therefore cannot make any conclusions on the frequency of *BCL2* translocation-negative FL. However, our study does suggest that a proportion of lymphomas regarded as *BCL2* translocation-negative FL are in fact NMZLs. Because NMZL is often a diagnosis of exclusion, this suggestion is currently hard to prove. However, previous studies suggest that *BCL2* translocation-negative FLs are genetically different from FLs with a *BCL2* translocation and have at least some features of NMZL. One study reported specific features of MUM1-positive and CD10-negative follicular lymphomas, including a frequent lack of *BCL2* translocations [4]. The combination of CD10 negativity and MUM1 positivity, both features of NMZL, and the lack of *BCL2* translocations suggest that this series might very well include NMZLs. A comparative genomic hybridization and gene expression study in *BCL2* translocation-negative FLs demonstrated less frequent expression of CD10 and enriched NF-κB signatures, both features of NMZL [7]. This suggests that (some of) these cases really are NMZLs, although the authors raise arguments against this, including strong BCL6 expression in all cases. We have however convincing data that NMZL cells can acquire BCL6 expression upon entering the follicular dendritic cell environment (manuscript in preparation). In a more recent comparative genomic hybridization study, translocation-negative FL showed aberrations that clustered more with NMZL than with FL with a *BCL2* translocation [8].

To finally resolve these issues, a positive marker for NMZL is highly needed. In addition to proposed new immunohistochemical markers for NMZL [9–11], better insight into its pathogenesis is essential for its proper classification. Here, a complicating factor is the fact that NMZL is often a diagnosis of exclusion, and therefore we cannot be sure that it represents a single entity. This possible heterogeneity might also be an impediment for studies into the pathogenesis of NMZL.

In conclusion, we have established immunomorphological characteristics of a group of low-grade B cell lymphomas that lacked a *BCL2* translocation. These criteria largely overlapped with the features of NMZL, and we propose that a subset of lymphomas that is currently often classified as FL without a *BCL2* translocation is actually better classified as NMZL.
